# Edge Temporal Digital Twin Network for Sensor-Driven Fault Detection in Nuclear Power Systems

**DOI:** 10.3390/s25247510

**Published:** 2025-12-10

**Authors:** Shiqiao Liu, Gang Ye, Xinwen Zhao

**Affiliations:** 1 College of Nuclear Science and Technology, Naval University of Engineering, Wuhan 430033, China; liusq02@cnnp.com; 2 China Nuclear Power Operation Technology Corporation, Wuhan 430233, China; 3 School of Computer Science, Wuhan University, Wuhan 430072, China; yeg@whu.edu.cn

**Keywords:** nuclear power system, edge computing for sensor data, sensor-based digital twin, fault detection

## Abstract

The safe and efficient operation of nuclear power systems largely relies on sensor networks that continuously collect and transmit monitoring data. However, due to the high sensitivity of the nuclear power field and strict privacy restrictions, data among different nuclear entities are typically not directly shareable, which poses challenges to constructing a global digital twin with strong generalization capability. Moreover, most existing digital twin approaches tend to treat sensor data as static, overlooking critical temporal patterns that could enhance fault prediction performance. To address these issues, this paper proposes an Edge Temporal Digital Twin Network (ETDTN) for cloud–edge collaborative, sensor-driven fault detection in nuclear power systems. ETDTN introduces a continuous variable temporal representation to fully exploit temporal information from sensors, incorporates a global representation module to alleviate the non-IID characteristics among different subsystems, and integrates a temporal attention mechanism based on graph neural networks in the latent space to strengthen temporal feature learning. Extensive experiments on real nuclear power datasets from 17 independent units demonstrate that ETDTN achieves significantly better fault detection performance than existing methods under non-sharing data scenarios, obtaining the best results in both accuracy and F1 score. The findings indicate that ETDTN not only effectively preserves data privacy through federated parameter aggregation but also captures latent temporal patterns, providing a powerful tool for sensor-driven fault detection and predictive maintenance in nuclear power systems.

## 1. Introduction

Nuclear power, compared to other traditional energy sources, operates in more extreme physical environments, requiring the deployment of numerous industrial sensors to monitor operational states and perform early fault detection. These sensors transmit and store sampled data in the form of time series, which are then communicated to the data center via gateway communication for analysis [1]. In a nuclear power digital twin system, the entire physical nuclear power system (GS) is equipped with a virtualized twin system (VGS), which performs high-precision mirror simulation of the entire system at the data and data association levels [2]. We assume that the simulation accuracy is infinitely close to the real physical system, expressed as $VGS\overset{sim\left(*\right)}{\to }GS$, where VGS represents the digital twin of the power generation system, GS represents the physical-world entity of the power generation system, and the function *sim*(*) represents the digital twin model with * denoting the input information required by the model. The purpose of building a digital twin for the nuclear power system is to make the digital twin system infinitely close to the real physical system so that it can anticipate latent failures and intervene in time to ensure the normal operation of the physical system [3].

Current research focuses on alleviating data heterogeneity and non-independent and identically distributed (non-IID) data caused by the diversity of edge devices while reducing the interaction load between the two systems [4]. We found that these mitigation measures fail to provide the digital twin with additional effective information because power monitoring data is still treated as static within the twin system. However, edge devices in nuclear power systems generate a large amount of time series data, such as the turbine speed of nuclear power reactors, and the temperature changes in reactor cooling water. The digital twin system should fully exploit the rich information contained in these data in the temporal domain.

Nevertheless, implementing this cloud–edge collaborative digital twin model in practice is challenging. Due to privacy and security concerns, monitoring data from different nuclear power production departments are typically restricted from centralized collection or sharing [5]. Additionally, physical differences among power equipment of various models or brands cause the generated data to deviate from the independent and identically distributed (IID) assumption [6]. These issues pose new challenges for constructing effective nuclear power digital twin models.

Therefore, the core research problem addressed in this work is how to construct a digital twin framework that can effectively model temporal dependencies and perform fault detection under the dual constraints of data privacy and non-IID distributions. Most existing approaches either ignore temporal dynamics by treating sensor data as static or assume centralized data availability, which is unrealistic in nuclear power scenarios. To overcome these limitations, this study proposes the ETDTN, a privacy-preserving and time-aware framework that enables cloud–edge collaborative learning for sensor-driven fault detection in nuclear power systems.

This setting represents a typical cloud–edge collaborative scenario in digital twin systems, where data from each nuclear power facility are retained locally and differ in distribution. Under these constraints, the proposed model is trained at the network edge and aggregated through the cloud to achieve a more generalized global digital twin representation for nuclear power systems.

**Figure 1:**
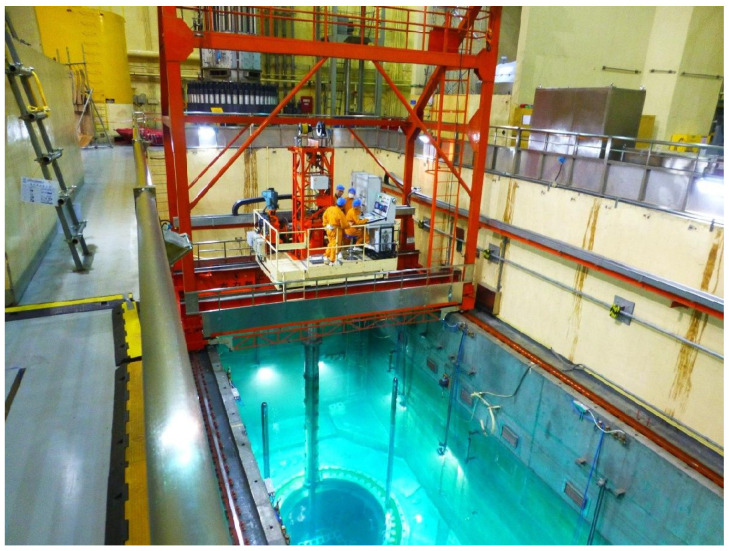
Staff inspecting anomalies above the reactor through the local digital twin. (source: China Nuclear Power Operation Technology Corporation, Wuhan, China).

As shown in Figure 1, a large number of sensors deployed in the nuclear reactor construct a digital twin system, enabling nuclear power plant staff to examine local anomalies based on the data provided. However, due to safety and confidentiality requirements, these data are typically not shared in real time across different nuclear departments. As a result, each local digital twin lacks external information, limiting its ability to discriminate and generalize against complex and variable anomaly patterns.

To address this, we propose an Edge Temporal Digital Twin Network for training large-scale digital twin models of nuclear power systems. ETDTN relies on the organic integration of three key components to form a set of neural networks that train edge data from nuclear power networks, ultimately enabling fault detection in nuclear power systems. The primary innovations of ETDTN are as follows:

- Continuous variable temporal representation. We introduce a novel continuous-variable time series modeling approach, in which the input data are no longer static or uniformly sampled but represented as continuous non-equidistant temporal variables. Time is incorporated as a probabilistic condition in variational inference, enabling the digital twin to learn temporal dependencies beyond discrete sampling intervals. This provides a theoretical advancement in representing irregular temporal dynamics for sensor-driven systems and significantly enhances time-domain generalization.
- Global representation-based digital twin module. A new global representation module is designed to mitigate the non-independent and identically distributed (non-IID) nature of nuclear power data. Through cloud–edge collaboration, each subsystem independently trains its local model and contributes to a unified global digital twin without data sharing. This design realizes privacy-preserving global knowledge transfer across heterogeneous nuclear subsystems.
- Temporal attention representation mechanism. We further propose a temporal attention mechanism embedded within a graph neural network in the latent space, which for the first time models temporal evolution as graph evolution. This mechanism allows ETDTN to capture non-Euclidean temporal dependencies between historical and future states, offering stronger interpretability and generalization than conventional self-attention architectures.

## 2. Related Work

In the field of nuclear power fault detection, early methods focused on predicting the Remaining Useful Life (RUL) of individual components within the system [7]. By forecasting faults in advance, these methods enabled preventive interventions, ensuring the safe and efficient operation of nuclear power production activities and preventing various unexpected situations. However, such methods could not effectively leverage global data from multiple devices or even multiple departments for correlated fault analysis. This limitation led to the emergence of machine learning-based fault detection methods, which can be categorized into two types: model-based methods and data-driven methods.

Model-based methods typically rely on expert prior knowledge to construct a digital twin model that closely aligns with reality. These models are then used to predict the degradation of new samples. The discrepancies between predicted and actual results are used to further refine the model. Examples of these methods include quadratic regression models [8], exponential models [9], Archard wear models [10], and Lemaitre damage models [11].

Data-driven methods, on the other hand, aim to minimize the reliance on prior knowledge. They train digital twin systems directly using large amounts of data to develop prediction models with generalization capabilities [12]. Research on data-driven fault detection methods can be divided into two categories. The first category is the Health Indicator (HI) method [13], which constructs a realistic HI to reflect the wear characteristics and patterns of industrial equipment. Faults are then predicted based on the latent correlation between HI and the remaining useful life of the equipment. The second category involves the use of machine learning algorithms to directly predict the status of industrial equipment in an end-to-end manner. For example, studies [14,15,16] directly use raw bearing data to predict their remaining service life.

While model-based approaches provide strong interpretability through physical priors, they heavily rely on expert knowledge and often fail to capture complex nonlinear degradation behaviors in large-scale sensor networks. Conversely, data-driven methods can automatically learn fault patterns from raw data, but their performance strongly depends on data quality and distribution assumptions, making them less reliable under privacy and non-IID constraints in nuclear systems.

However, machine learning-based methods impose high requirements on the quality of raw data, especially regarding the independent and identically distributed (IID) property, which is challenging to meet in nuclear power monitoring data [17]. Data from different nuclear power facilities often follow their own unique distributions, and data sharing across different departments may be restricted. These limitations pose new challenges for fault detection tasks in nuclear power systems.

Recent digital-twin studies have further pushed the paradigm toward data-centric and distributed settings. A first direction integrates high-fidelity simulation models with online sensor streams to support predictive maintenance in energy and process industries, but still relies on centralized data access and stable sampling rates [18]. A second direction introduces learning-enhanced or AI-enabled twins, in which temporal encoders, graph neural networks, or attention mechanisms are embedded into the twin to capture cross-subsystem correlations; however, these approaches are typically evaluated on single-plant scenarios and do not consider strict privacy constraints [19]. A third and more relevant direction is edge/federated digital twins for industrial IoT, where model parameters are aggregated across sites to avoid raw-data sharing, yet time is usually treated as uniformly sampled and non-IID distributions across plants are not explicitly aligned. Therefore, even in the latest digital-twin frameworks [20,21], two issues remain insufficiently addressed for nuclear power systems: (i) representation of irregular, non-equidistant temporal signals, and (ii) mitigation of privacy-induced non-IID heterogeneity at the latent level. The proposed ETDTN is designed exactly to fill these two gaps.

In summary, although both model-based and data-driven approaches have advanced fault detection in nuclear systems, they remain limited in addressing privacy-preserving and non-IID data scenarios. Recent advances in federated learning, temporal representation learning, and graph neural networks provide new opportunities, but current works rarely combine these perspectives within a unified digital twin framework. Therefore, a comprehensive solution that simultaneously captures temporal dependencies and preserves data privacy is still missing, which motivates the development of the proposed ETDTN.

## 3. Edge Temporal Digital Twin Network

### 3.1. Digital Twin Autoregressive Training in Nuclear Power System

For the global digital twin model, the ideal scenario is the existence of a unified department with coordination capability that can aggregate sensor data from different subsystems and use these data to train and construct a digital twin model with strong generalization ability. However, in the field of nuclear power production, due to privacy and security restrictions at individual nuclear reactors, edge sensors cannot connect to a gateway that bypasses local limitations, and therefore the data from different units cannot be directly aggregated. The currently adopted solution is to utilize the federated learning paradigm for model parameter aggregation, that is, the data from local nuclear power plant sensors are processed only by local computational power to train models on site, and then the updated neural network parameters of the local models are uploaded to the cloud server. The cloud server collects the parameters from all edge models, aggregates them, and sends the updated parameters back to the local devices, thereby avoiding the collection of sensitive raw monitoring data. This process can be formalized as:

(1) $$\underset{\theta }{\min}F\left(\theta \right)=\sum_{i=1}^{N}p_{i}E_{\left(x,y\right)\sim D_{i}}f_{i}\left[\rho_{i}\left(\theta ;x\right),y\right]$$

where *F*(*θ*) represents the loss function of the global model with *θ* as parameter on the cloud server, and *p_i_* represents the proportion of the number of samples of the *i*-th client to the number of samples of all clients. *D_i_* represents the distribution obeyed by the *i*-th client sample, and $f_{i}\left[\rho_{i}\left(\theta ;x\right),y\right]$ represents the loss function generated by the *i*-th client under the local model $\rho_{i}\left(\theta ;x\right)$ mapping. *x* represents the input sample, *y* represents the label, and *N* represents the number of edge clients in the cloud-edge collaborative network. It can be found that under this federated learning paradigm, each client can deploy its own local model *ρ*, and after multiple rounds of iterations locally, the updated parameters are uploaded to the cloud server for aggregation. However, it can be observed that the current time information is not included in the entire computation, and this process ignores the latent information of the sample in the time domain. In order to utilize and characterize the latent time-domain patterns contained in the data, we define the input sample in the form of a time series, and the input window of the local model i is read in a sliding manner on *x_i_* (*t*) in temporal order. Assuming that the width of the reading window is *w_i_,* the input sample of the data at time *t_k_* is $x\left(t_{k}:t_{k+w_{i}}\right)$.

At this time, the cloud-edge system training process can be formalized as:

(2) $$\underset{\theta }{\min}F\left(\theta ,t\right)=\sum_{i=1}^{N}p_{i}E_{\left(x,y\right)\sim D_{i}}f_{i}\left\{\rho_{i}\left[\theta ;x_{i}\left(t_{k}:t_{k+w_{i}}\right)\right],y_{i}\right\}$$

where $F\left(\theta ,t\right)$ represents the loss function of the global model with *θ* as parameter on the cloud server at time *t*. $x_{i}\left(t_{k}:t_{k+w_{i}}\right)$ represents the sample with input length *w_i_* at time *t_k_*, and *y_i_* represents the corresponding label. In reality, power equipment usually has no labels at each time interval. In the digital twin framework, the data at future time can be set as the training label of the current sample, thereby constructing a self-supervised representation learning framework under the digital twin paradigm. In Formula (2), when the *i*-th client input sample is $x_{i}\left(t_{k}:t_{k+w_{i}}\right)$, let $y_{i}=x_{i}\left(t_{k+w_{i}}+t_{k+w_{i}+\lambda_{i}}\right)$, then Formula (2) is transformed into a digital twin autoregressive learning framework, and its label *y* is replaced by the real sample with future time length *λ_i_*. The entire global model is transformed into the form of digital twin autoregressive training, and the local device directly uses the sample at the future *λ_i_* time as the supervision signal for model training.

### 3.2. Mapping of Edge Time Series Samples

For a time series sample at the edge, we hope to find a mapping *ρ_i_* that satisfies

(3) $$\rho_{i}:\;x\left(t_{k}:t_{k+w_{i}}\right)\to \hat{x}\left(t_{k}:t_{k+w_{i}+\lambda_{i}}\right)$$

where *ρ_i_* represents the local model of the *i*-th power department, which maps a series of samples at time $t_{k}:t_{k+w_{i}}$ to the estimated value after time *λ_i_*. Here, the time span *λ_i_* is a continuous variable. This setting is very critical for the training of cloud-edge collaborative time series models.

First, the sample timestamps collected by different departments are different, and the continuous time span allows each local model to continuously adjust the future-oriented time span according to its own sample time, ensuring the consistency of sample time when each edge communicates with the digital twin.

Second, the power monitoring data collected locally does not necessarily meet the independent and identically distributed properties. The key latent patterns contained in the data in the time domain may exist in multiple time scales of different distributions. The continuous time span variable gives the edge model a certain degree of freedom in time series representation. In the local training stage, the edge model can input samples of any time span as self-supervision signals as needed to improve the generalization performance of the global digital twin model in the time domain.

In order to obtain a universal global representation model in the digital twin system, it is first necessary to define the local model training method of the client. Usually, the input samples of neural network training need to meet the requirements of being fully observed and fixed-size. As shown in Equation (3), the span of future time *λ_i_* is a variable continuous value, which means that the input samples are non-equidistant. The local model needs to adapt to this non-equidistant property to implement training. The hardware of each power device usually lacks the basic computing power for deep learning, but they can transfer the collected data to a local device of the power department with basic computing power. For example, micro-meteorological sensors can upload the collected data to the substation to which they belong, and the substation provides computers to process the data. The data source is the collected time series data and timestamps. The function of the local model is to use historical data as input and samples at future time points with variable spans as supervision signals to pre-train a local model with predictive performance. This process can be expressed as:

(4) $$\max\log P\left[x\left(t_{k}:t_{k+w_{i}+\lambda_{i}}\right)|\;x\left(t_{k}:t_{k+w_{i}}\right)\right]$$

(5) $$\lambda_{i}=\left\lceil h_{i}\right\rceil ,\;h_{i}\sim N\left(0,\tau^{2}\right)$$

where *h_i_* represents a random time increment, which can be negative. This is because ETDTN uses samples as self-supervisory signals to participate in the training of the local model, so only the original samples of the corresponding time need to be input. The standard deviation *τ* is used as a hyperparameter to adjust the prediction time range of the local model. Increasing *τ* can enable the model to select supervisory signals in a wider time range and enhance long-distance generalization. However, since the stability of the data decreases over a longer time span, the representation accuracy of the model will decrease accordingly.

It is difficult to directly solve Equation (4) because the probability *P* contains the conditional prior of the continuously variable time parameter. The previous method of using Bayesian approximation fails here. We use the basic framework of variational Bayesian expectation maximization for derivation. Assuming that the input sample starts at time 0, that is, *t* = 0, we have:

(6) $$\begin{array}{l} \log P\left[x\left(t_{k+w_{i}}:t_{k+w_{i}+\lambda_{i}}\right)|\;t_{1}:t_{k+w_{i}+\lambda_{i}},x\left(t_{1}:t_{k+w_{i}}\right)\right]\\ =\int_{z}q\left[z\;|\;x\left(t_{k+w_{i}}:t_{k+w_{i}+\lambda_{i}}\right),t_{1}:t_{k+w_{i}+\lambda_{i}},x\left(t_{1}:t_{k+w_{i}}\right)\right]\cdot \\ \log p\left[x\left(t_{k+w_{i}}:t_{k+w_{i}+\lambda_{i}}\right)|\;t_{1}:t_{k+w_{i}+\lambda_{i}},x\left(t_{1}:t_{k+w_{i}}\right)\right]dz\\ =\int_{z}q\left[z\;|\;x\left(t_{1}:t_{k+w_{i}+\lambda_{i}}\right),t_{1}:t_{k+w_{i}+\lambda_{i}}\right]\cdot \\ \log\frac{p\left[z,x\left(t_{k+w_{i}}:t_{k+w_{i}+\lambda_{i}}\right)|\;t_{1}:t_{k+w_{i}+\lambda_{i}},x\left(t_{1}:t_{k+w_{i}}\right)\right]}{q\left[z\;|\;x\left(t_{1}:t_{k+w_{i}+\lambda_{i}}\right),t_{1}:t_{k+w_{i}+\lambda_{i}}\right]}dz+\\ D_{KL}\left\{q\left[z\;|\;x\left(t_{1}:t_{k+w_{i}+\lambda_{i}}\right),t_{1}:t_{k+w_{i}+\lambda_{i}}\right]\|\;p\left[z\;|\;x\left(t_{1}:t_{k+w_{i}+\lambda_{i}}\right),t_{1}:t_{k+w_{i}+\lambda_{i}}\right]\right\} \end{array}$$

where *z* is a latent variable. It represents the hidden state of the digital twin in the latent space, capturing temporal evolution patterns across non-equidistant sensor observations. The conditional time term *t* acts as a continuous constraint that links each sample to its temporal context. During training, the ELBO is optimized through the standard reparameterization trick, where *z* = μ + σ ⊙ ε, and ε ~ *N*(0,1). Monte Carlo sampling is used to approximate the expectation term, ensuring stable and efficient gradient propagation. *q* represents the probability of inferring *z* from historical data and future time. It is the distribution induced by expanding Equation (6) with respect to the integral variable *z*. In Equation (7), a KL-divergence is also separated. Since this term is greater than 0, Equation (7) can be scaled to have:

(7) $$\begin{array}{l} \log p\left[x\left(t_{k+w_{i}}:t_{k+w_{i}+\lambda_{i}}\right)|\;t_{1}:t_{k+w_{i}+\lambda_{i}},x\left(t_{1}:t_{k+w_{i}}\right)\right]\\ \geq \int_{z}q\left[z\;|\;x\left(t_{1}:t_{k+w_{i}+\lambda_{i}}\right),t_{1}:t_{k+w_{i}+\lambda_{i}}\right]\cdot \\ \log\frac{p\left[z\;|\;x\left(t_{1}:t_{k+w_{i}}\right),t_{1}:t_{k+w_{i}}\right]\prod_{j=1}^{\lambda_{i}}p\left[x\left(t_{w_{i}+j}\right)|\;t_{w_{i}+j},z\right]}{q\left[z\;|\;x\left(t_{1}:t_{k+w_{i}+\lambda_{i}}\right),t_{1}:t_{k+w_{i}+\lambda_{i}}\right]}dz\\ =E_{z\sim q\left[z|X\left(t_{1}:t_{k+w_{i}+\lambda_{i}}\right),t_{1}:t_{k+w_{i}+\lambda_{i}}\right]}\left\{\sum_{j=1}^{\lambda_{i}}\log p\left[x\left(t_{k+w_{i}+j}\right)|\;t_{k+w_{i}+j},z\right]\right\}-\\ D_{KL}\left\{q\left[z\;|\;x\left(t_{1}:t_{k+w_{i}+\lambda_{i}}\right),t_{1}:t_{k+w_{i}+\lambda_{i}}\right]\|\;p\left[z\;|\;x\left(t_{1}:t_{k+w_{i}}\right),t_{1}:t_{k+w_{i}}\right]\right\} \end{array}$$

It can be found that Equation (7), obtained after scaling Equation (6), is simplified into two parts. The first part is an expectation term, which does not have a direct analytical form, but can be approximated by a neural network. The second part is a KL divergence. When the specific distribution form of the intermediate variable is specified, the analytical formula of the term can be obtained, so it can directly participate in the optimization process. Compared with the conventional Bayesian expectation maximization method, Equation (7) is significantly different, that is, the intermediate latent variable *z* needs to obey a conditional probability distribution, and the conditional part of this distribution includes the time to predict the target, which means When designing a digital twin system, in addition to the value of the current time series data, the input of the model also needs the time of the current data and the time in the future direction, where the time in the future direction ends at the timestamp of the sample in the supervision signal.

According to Equation (7), we designed the specific structure of ETDTN to realize collaborative training of local power monitoring data.

**Figure 2:**
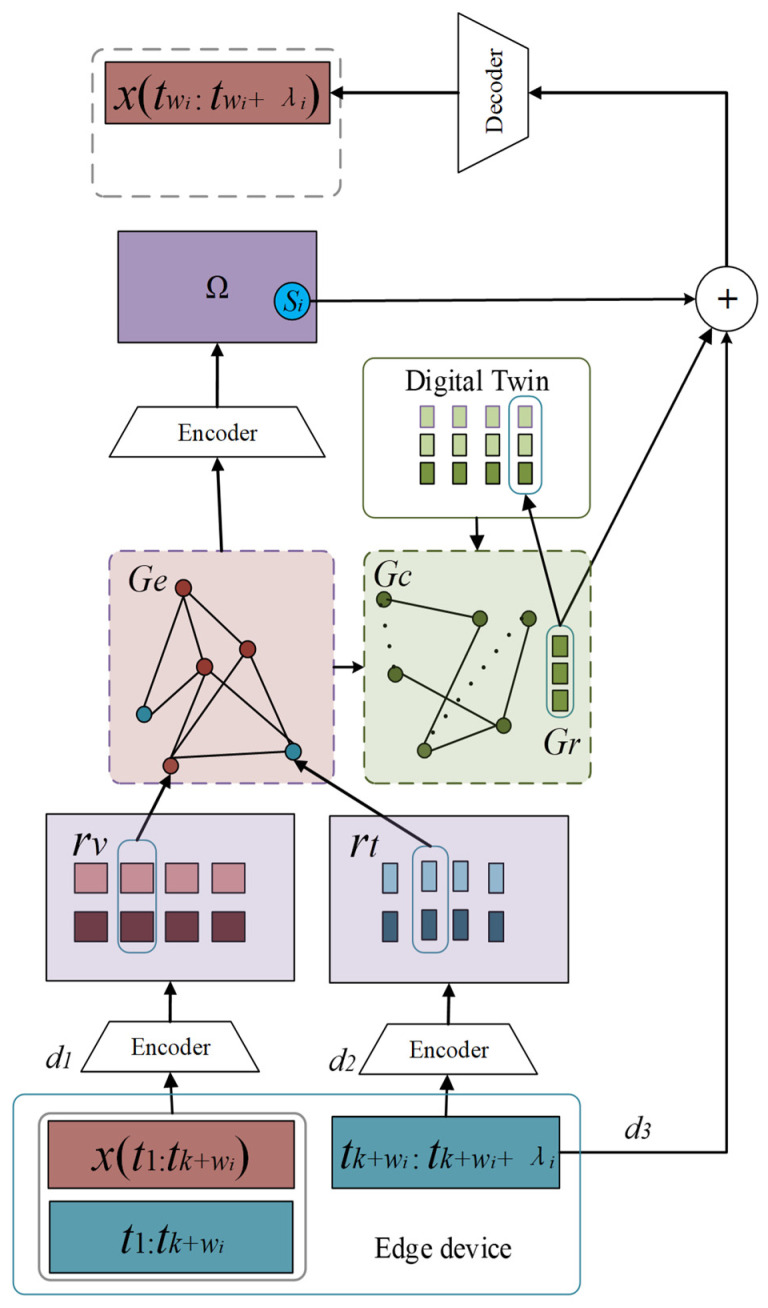
Edge Temporal Digital Twin Network.

As shown in Figure 2, the input information of ETDTN comes from edge power equipment. The input information is divided into two parts. The first part is the historical sample $x\left(t_{1}:t_{k+w_{i}}\right)$ and the corresponding time $t_{1}:t_{k+w_{i}}$. The second part is the time of the sample to be predicted, which is related to the self-predicted sample. The timing of supervision signals remains consistent. These data are introduced into ETDTN through three different paths and are represented by a graph neural network in the latent space. The graph neural network shares the parameters of the graph with the nuclear power system digital twin and constrains the representation information of edge devices through the graph attention mechanism toward the future direction. And, for the global direction evolution, the representation information is finally decoded by the decoder, and the predicted sample value is output. The purpose of training ETDTN is to obtain the digital twin representation *Gr* and the parameters in the graph *G_C_* located in the latent space. They not only contain the graph association information of each edge power equipment, but also include their time information patterns, which will facilitate the docking of multiple downstream fault detection tasks.

As shown in Figure 2, the input information of ETDTN comes from edge power equipment and is divided into two parts. The first part is the historical sample *x*(*t*_1_*:t_k_ + w_i_*) and its corresponding time *t*_1_*:t_k_ + w_i_*; the second part is the time of the sample to be predicted. In the autoregressive training with non-equidistant sampling, this time is consistent with the timing of the supervision signals. These data are input into ETDTN through three paths (*d*_1_, *d*_2_, and *d*_3_) and represented in the latent space by a graph neural network *Ge*. Through graph attention transformation, *Ge* evolves toward the future direction into graph *Gc*. *Gc* shares its parameters with the digital twin of the nuclear power system and constrains the representation information of edge devices through the graph attention mechanism oriented toward the future. The decoder receives the future-directed information transmitted from the three paths. The first information is the sample from the same-distribution probability space Ω generated by *Ge*. The second information is the digital twin vector *Gr* generated by *Gc*. The third information is the time of the sample to be predicted, directly transmitted through the d3 path. These pieces of information are fused together through vector concatenation and finally decoded by the decoder to output the predicted sample value.

The purpose of training ETDTN is to obtain the digital twin representation information and the parameters of the latent graph located in the latent space. They not only contain the graph association information of each edge power device but also their temporal information patterns, which facilitate the integration and application of multiple downstream fault detection tasks. The information transmission process shown in Figure 2 will be described in detail in Section 3.3, Section 3.4 and Section 3.5.

### 3.3. Input and Encoding of Edge Device Samples

Information is injected into ETDTN through three paths. The sample values and corresponding timestamps read from the input window of length *w_i_* are passed into the model through path d_1_, corresponding to the time in the window of the first and second conditions in the conditional distribution of the expected intermediate variable *z* in Formula (7). The timestamp of the sample to be predicted is passed into the model separately through path d_2_, corresponding to the time outside the window in the second condition in the conditional distribution of the expected intermediate variable *z* in Formula (7). These two paths pass information into their respective encoders for encoding. Here, the encoder is a neural network interface, and different neural network structures can be used as needed. This paper uses a fully connected neural network by default. In addition, the sample time to be predicted is also passed directly to the input of the decoder through a d_3_ path. This is to ensure the data still have strong temporal shallow semantic information in the decoding stage, so as to constrain ETDTN to output in the temporal direction of the supervision signal.

### 3.4. Cloud-Edge Collaborative Temporal Attention Representation

The sample and future time information are encoded and then passed into the latent space to form the sample representation *r_v_* and future time representation *r_t_* respectively. Then each column of them is passed into the graph neural network *Ge* as a latent representation and becomes a node on *Ge*. The introduction of the latent representation information of the sample and the latent representation information of the future time in the same graph is to allow the sample information to be updated under the guidance of the future time, and the actual samples in the future time also happen to participate in the training process of the model as self-supervisory signals. In this way, the model establishes a non-Euclidean pattern association for historical samples and future time in the latent space. Define the nodes of the graph neural network *Ge*:

(8) $$V=\left\{v_{k}\;|\;v_{k}\in r_{v}\cup r_{t}\right\}$$

where *V* represents the set of *Ge* nodes, and *v_k_* represents the *k*-th node on the graph. The weight of the edge between nodes represents the strength of the association between the nodes. In the initialization stage of *Ge*, all nodes are set to be connected by edges, and the weight of each edge is initialized to 1. The lower limit threshold of the edge is set to 0 < γ < 1. In the training stage, as the gradient updates the network parameters, when the weight of the edge is lower than γ, the edge is deleted. The nodes of *Ge* contain all sample information and time information of the current local device, as well as the time information of the sample to be predicted. Attention updating of each edge of *Ge* can redistribute the original one-dimensional time series information structure on the graph, realizing the representation of the time series pattern in high-dimensional non-Euclidean space. In order to establish connections between points in *Ge*, we take any two nodes $v_{i},v_{j}\in V$ and define the edge between them as

(9) $$E\left(v_{i},v_{j}\right)=\frac{\exp\left\{\sigma \left[a_{ij}^{T}\cdot \left(W_{i}\left(v_{i}⊕v_{c}\right)⊕W_{j}v_{j}⊕W_{c}v_{c}\right)\right]\right\}}{\sum_{k=1}^{n_{1}+n_{2}}\exp\left\{\sigma \left[a_{ij}^{T}\cdot \left(W_{i}\left(v_{i}⊕v_{c}\right)⊕W_{j}v_{k}\right)\right]\right\}}$$

In Formula (9), $E\left(v_{i},v_{j}\right)$ represents the weight of the edge between nodes *v_i_* and *v_j_*. *σ* represents the nonlinear transformation function, $W_{i}\in R^{m\times 2n}$ and $W_{j}\in R^{m\times n}$ are the left multiplication matrices corresponding to *v_i_* and *v_j_* respectively, and the elements in the matrices are learnable parameters. Through this linear transformation, the dimension of the node in the graph is compressed to m. When the parameters of the graph are updated, the *w_i_* matrix adjusts the association strength between the sample latent information and the future time information. The symbol ⊕ represents the concat operation, which is used to connect two vectors. *a_ij_* represents the attention weight, which is used to adjust the attention weight between nodes. *v_c_* comes from the node that records the future time information. When *i* = *c*, the future time node of *v_i_* is itself.

When the graph is updated, the weight *w_i_* adjusts the weight of this target information so that the sample information on the graph is associated with the future time information. For each node in *Ge*, we use the collaborative temporal attention mechanism to update.

(10) $$v_{i}^{\prime }={\left\{1+\exp\left(-\sum_{k=1}^{n_{1}+n_{2}}e\left(v_{i},v_{k}\right)W_{i,k}v_{i}\right)\right\}}^{-1}$$

where *W_i,k_* represents the linear transformation matrix of the edge between *v_i_* and *v_j_*. It can be found that the update of $v_{i}^{\prime }$ uses its attention information with all other nodes, so each node in the updated graph *G_C_* contains sample information, sample time and future time information at the same time. The future time information is embedded in each updated node, making the node continuously variable in future time semantics.

Each node of the updated graph *G_C_* forms an edge graph representation vector *G_r_*, which carries the information of the edge device at the time of this graph update. This information is transmitted to the cloud server through interaction with the power digital twin. The cloud server averages these information edge graph representation vectors and sends the averaged vector to the edge graph *G_C_*, thereby guiding the next round of cloud-edge timing attention update. In this process, any round of edge power equipment can obtain global timing information, and this information is implemented by the graph attention update mechanism.

### 3.5. Decoding Stage

In order to make the representation of ETDTN more generalizable under different data distributions, the information of the embedded graph Ge will not only evolve into Gc through the attention mechanism, but will also be directly encoded as the latent distribution parameters of the input information on another path, thereby determining a specific latent distribution corresponding to the local power equipment in the probability space Ω. ETDTN randomly samples the distribution and then concatenates the sample *s_i_* with the current edge graph representation vector Gr and the time of the d3 path to form an aggregated decoding vector. After the vector is passed to the decoder, the sample estimate corresponding to the future time is output $\hat{x}\left(t_{k+w_{i}}:t_{k+w_{i}+\lambda_{i}}\right)$. That is:

(11) $$Adv_{i}=S_{i}⊕Gr⊕d_{3}\left(t,\lambda_{i}\right)$$

where *Adv_i_* represents the aggregated decoding vector finally passed to the decoder, *S_i_* represents the sampled latent representation sample, $d_{3}\left(t,\lambda_{i}\right)$ represents the information transmitted on the *d*_3_ path, the parameter t represents the starting time point of the future sample, and *λ_i_* represents the time span. The sampling information in the aggregated decoding vector is consistent with the sampling process of VAE. This branch path gives ETDTN a certain generation ability, making the output information more diverse and enhancing the generalization ability of the model. The *d*_3_ path is the future time information. As the output of the decoding process, it directly constrains the shallow time semantics, so that the model still has stronger temporal semantic guidance in the decoding stage. The decoded information is compared with the supervisory signal $x\left(t_{k+w_{i}}:t_{k+w_{i}+\lambda_{i}}\right)$ to solve the LOSS minimization, and then the digital twin is trained for autoregression. When the training is completed, the global digital twin model has a certain degree of general representation ability, and it can be connected to various types of downstream tasks. In power grid equipment, fault detection has always been an important task. Therefore, this study applies ETDTN to fault detection of power equipment to test its prediction performance.

## 4. Experiment

This dataset comprises data from 17 independent units sourced from the China Nuclear Power Industrial Internet Platform, and the information is organized into four subsystems: core data (CD), thermal cycle data (TC), turbine system data (TS), and power output data (PO), where the core system contains 22 attributes, the thermal cycle system contains 35 attributes, the turbine system contains 24 attributes, and the energy storage system contains 8 attributes. For a fair comparison, all baseline methods were deployed under the same 17-client cloud–edge federated setting. The original dataset was partitioned by nuclear unit into 17 disjoint clients, and each client retained its own 80%/20% train–test split, so that all methods were exposed to the same non-IID distribution. In the dataset, fault-labelled intervals correspond to known incident periods in the plant’s operation; however, explicit fault types (for example, control-rod jamming, coolant flow reduction, turbine-blade fatigue, or generator excitation failure) are not consistently annotated. Instead, we regard any time-series record within one of these labelled fault intervals as containing fault-related deviations, as manifested by the monitoring data exceeding a predefined threshold ε (mean square error between predicted and actual values). This labelling scheme enables detection of incipient and subtle anomalies even in ultra-low-fault-rate nuclear-power conditions, where the occurrence of clearly defined fault events is rare.

The experiment assumes that these 17 units do not share information with each other. Therefore, each unit, as an edge node, independently trains its own local model and uploads the training parameters to the global digital twin during each iteration. We classify the data according to the type of nuclear power plant and the fault rate range of each type to verify the fault prediction accuracy of ETDTN under different fault occurrence rates in real equipment. We define the fault criterion as the mean square error (MSE) between the predicted data and the actual data. When the MSE exceeds the threshold ε, the model is considered to have failed. The experimental hardware used includes an Intel i7-14700KF CPU and an NVIDIA RTX3090 24 G GPU. Deep learning frameworks were deployed on PyTorch 2.3. The optimizer used is ADAM [22], with exponential decay factors for the first-order and second-order moments set to 0.85 and 0.997, respectively, and the learning rate set to 0.0012, *ε* is set to 0.001. Each edge dataset was split into 80% for training and 20% for testing. The dataset contains 2,557,871 time-series records: CD—521,842, TC—892,451, TS—706,305, and PO—437,273.

The comparison methods in the experiment include: Prophet [23], which first decomposes the time series data and then models the power time series data using fitting methods; GRU-D [24], based on the gated recurrent unit (GRU) neural network [25], which decomposes the input into three parts: variables, masks, and time series segments, enabling the model to better infer distribution changes; VAE-RNN [26], a variational autoencoder based on a recurrent neural network; ODE-RNN-ODE [27], based on neural ordinary differential equations, which constructs encoders and decoders to achieve continuous representation of samples; Informer [28], which uses a self-attention mechanism to improve the model’s ability to predict long-term dependencies while reducing computational complexity by adjusting the scope of the attention mechanism; and Autoformer [29], which replaces the attention mechanism with an autocorrelation mechanism to further enhance the model’s predictive performance. Since Informer and Autoformer were originally developed for centralized long-sequence forecasting, we wrapped their encoder–decoder architectures into the same FedAvg-style training loop as Equation (1), where local parameters are updated on each client and then synchronously aggregated on the server. Optimizer, learning-rate order, batch size, and the number of local epochs per round were aligned across methods whenever the model architectures allowed. The evaluation metrics used in the experiment are mean square error (MSE) and F1 score.

### 4.1. Fault Detection

The experimental results are shown in Table 1. ETDTN has achieved the best fault detection performance in each fault rate scenario in each power sector. It can be found that ETDTN can achieve better results when the inherent fault rate of the scenario is smaller. This reflects the advantage of the ETDTN digital twin system, that is, when the physical environment is low-fault, the twin system is also in a low-fault state, so the model’s misjudgment rate is lower than other baseline methods. The digital twin system here becomes a copy of the edge device in the cloud, which corresponds to the conditional part in the likelihood estimation. In this state, the model has higher precision, resulting in a leading F1 score. At the same time, the experimental results also show that directly adding the self-attention mechanism on the encoder side is not the best choice, because it not only increases the computational time, but also the fault detection performance is not as good as ETDTN that deploys attention on the latent graph.

**Figure 3:**
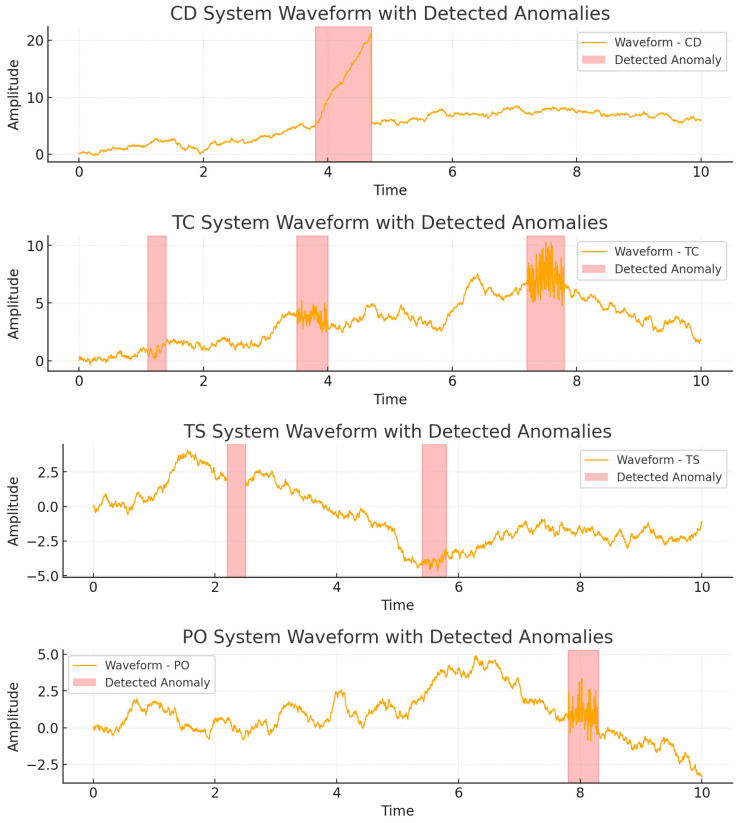
Prediction results of ETDTN on four subsystems of nuclear power.

As shown in Figure 3, the predictive performance of our model on the anomaly regions across four different subsystems of nuclear power can be intuitively observed. Anomalies in all four subsystems were detected (corresponding to the translucent intervals in the figure). Some anomalies can be directly identified from the waveform’s morphological changes, as they exhibit significant pattern differences from the surrounding context, while others are not easily noticeable yet are still detected by the model.

### 4.2. Ablation Experiment

We take the power station scenario as an example and conduct an ablation experiment on ETDTN to test the contribution of each component to the fault detection performance. The main components of ETDTN include: Temporal Attention Representation (TAR), digital twin module based on global representation (TW), continuous variable temporal representation (CVTR). To ensure the reproducibility of the ablation study in Table 2, the implementation of each variant is specified as follows. For the “−TAR” variant, the latent graph *Ge* was constructed with the same node set as in Section 3.4, but the attention-based edge update in Equations (9) and (10) was replaced by a simple averaging operation over the connected nodes. In this setting, no additional layers were introduced and the sizes of the encoder and decoder were kept unchanged; only the attention parameters were inactivated. For the “−TW” variant, the global representation-based digital twin module was removed, so that the client neither uploaded the latent graph representation *Gr* to the cloud nor received the aggregated global vector, and training was performed solely on the local latent graph. The remaining three-path encoding and decoding structures were retained to keep the parameter count comparable. For the “−CVTR” variant, the future time span *λ_i_* was fixed to 1 and the continuous-time conditioning *t* in Equations (6) and (7) was omitted, so that the model degraded to an equidistant time-series predictor while preserving the same encoder/decoder widths. In this way, the performance differences can be attributed to the removal of the corresponding component rather than to changes in model capacity.

In Table 2, the experimental results show that removing both TAR and TW has a significant negative impact on the fault detection performance of the digital twin. This indicates that both the graph attention evolution mechanism in the latent space and the global digital twin play a key role in the power data representation ability of ETDTN. The degradation is limited by removing CVTR, but it is about 15%, which indicates that the continuously variable form of time input does improve the generalization performance of the model. Ablation experimental results show that all three components of ETDTN play a key role in the performance of the model.

### 4.3. Parameter Analysis

The primary hyperparameter involved in ETDTN is the random time span standard deviation, *τ*, which is used to adjust the range of the time span λi (see Equation (5)). Empirically, when T is within a reasonable range, longer time spans force the model to achieve better long-range generalization. This section quantitatively analyzes the effect of this hyperparameter by setting different values of *τ* and verifying its impact on model performance. The experimental results are shown in Table 3:

As shown in Table 3, when *τ* = 0, the time span is fixed at 1 due to the mean sampling value being 1, making the variable time span non-functional. In this case, the model exhibits relatively low prediction accuracy, indicating that introducing a variable time span indeed helps the model learn distribution characteristics across various time distances, thus improving generalization performance. However, the results also show that when *τ* exceeds 0.75, model performance starts to decline. This is because beyond a certain standard deviation, the randomly selected next input sample may be too distant from the current one, leading to non-gradual changes in sample distribution and increasing statistical inconsistency across devices. Another hyperparameter is the lower bound threshold of the graph edges, γ, in the graph neural network Ge within the latent space. This threshold defines the minimum weight for edges in the graph neural network. When the correlation between nodes falls below this threshold, the edge between these nodes is removed to simplify the graph structure. Unlike *τ*, γ is less related to model performance but more relevant to computational efficiency. We observe the impact of γ on the model’s training efficiency by analyzing its convergence behavior.

### 4.4. Training Efficiency and Communication Overhead

To quantify the computational and communication cost introduced by the multiple encoders, the latent graph, and the temporal attention module, we further measured the average training time and per-round communication payload. We used the same federated setting as in Section 4, where 17 nuclear units participated in synchronous aggregation, and ran all methods on the same hardware platform to ensure fairness. We compared our ETDTN with two representative federated architectures, FedAvg and FedProx. The results are summarized in Table 4.

The results show that ETDTN introduces an additional 0.16–0.21 s per round compared with lightweight FL baselines. This overhead mainly comes from the latent space graph attention update (Section 3.4), while the three-path encoders only incur a small cost because they are implemented as fully connected networks. In terms of communication, the payload of ETDTN is about 13–17% higher than FedAvg/FedProx, since we transmit the graph-level representation parameters together with the model parameters. Considering that ETDTN consistently achieves higher fault-detection F1 scores under the same federated constraints (Table 1), we argue that this level of computational and communication overhead is acceptable for privacy-preserving nuclear power scenarios. Future work can further reduce the traffic by adopting sparse graph updates or parameter compression.

## 5. Discussion and Conclusions

The proposed Edge Temporal Digital Twin Network shows significant advantages in tackling the dual challenges of privacy restrictions and non-IID distributions that are intrinsic to nuclear power system monitoring. Unlike conventional federated learning frameworks that merely average parameters, ETDTN adopts a dual-channel mechanism: it not only aggregates local parameters but also integrates graph-based latent representations into the global model. This design allows edge units of nuclear power plants to retain sensitive operational data locally while still contributing to a global digital twin, aligning with the stringent privacy and security requirements of the nuclear industry.

Another innovation lies in its treatment of temporal information. Conventional digital twin approaches often assume equidistant and static time-series input, which neglects the non-uniform nature of sensor data in nuclear reactors. ETDTN introduces a continuous variable temporal representation, in which future timestamps are directly encoded into the model as conditional inputs. This allows the system to capture fine-grained temporal patterns such as gradual variations in turbine speed or cooling water temperature—patterns that are critical for early fault detection in low-fault-rate nuclear environments.

Furthermore, the temporal attention mechanism embedded within a graph neural network sets ETDTN apart from existing Transformer-based methods. By treating both historical samples and future timestamps as nodes in a latent graph, ETDTN establishes non-Euclidean associations between them. This enables evolutionary representation learning that mirrors the physical dynamics of reactor subsystems more faithfully, while reducing computational redundancy compared to standard self-attention models. The ablation study reinforces that TAR, the TW, and the CVTR are indispensable; removing any of them leads to sharp declines in fault detection accuracy, especially in core and turbine subsystems.

Compared with existing fault-diagnosis methods, ETDTN demonstrates clear and substantial advantages—both quantitatively and qualitatively. Quantitatively, as shown in Table 1, ETDTN achieves F1 scores of 92.01% in the CD subsystem at f < 0.001, 96.20% in the TC subsystem, 92.35% in the TS subsystem, and 89.27% in the PO subsystem—outperforming baselines such as GRU-D, VAE-RNN and Autoformer. Qualitatively, ETDTN introduces a continuous-variable temporal representation alongside a latent-graph attention mechanism, enabling enhanced extraction of fine-grained temporal features and improved generalization under non-IID and data-privacy constraints. In nuclear-power scenarios—where fault-rate data are scarce and system stability is paramount—ETDTN delivers sustained detection performance (F1 > 85%) even in ultra-low-fault-rate conditions (e.g., f < 0.05 in TS and PO). Therefore, the proposed strategy is not only more effective than existing methods but is also better suited for safety-critical nuclear applications.

Nevertheless, several challenges remain for deployment in industrial settings. Frequent parameter and representation updates could cause non-negligible communication overhead between nuclear facilities and cloud servers. Edge devices with limited computational capacity may also struggle to support complex temporal attention mechanisms. In addition, hyperparameters such as the standard deviation of the random time span introduce sensitivity that must be carefully tuned to different subsystems. Addressing these issues will be vital for scaling ETDTN to real-world nuclear power operations. Looking forward, the methodological innovations of ETDTN suggest a strong latent for broader applications in other safety-critical infrastructures such as smart grids and aerospace systems, where data privacy and temporal heterogeneity are equally pressing barriers.

In summary, this study developed and evaluated the Edge Temporal Digital Twin Network (ETDTN) as a novel cloud–edge collaborative framework tailored for sensor-driven fault detection in nuclear power systems. ETDTN introduces three unique components that distinguish it from conventional approaches: a continuous variable temporal representation that captures non-equidistant time dependencies, a global digital twin module that mitigates non-IID distributions without data sharing, and a temporal attention mechanism based on graph neural networks that enhances temporal feature learning in latent space. By combining these innovations within a privacy-preserving federated learning framework, ETDTN not only aligns with the operational constraints of nuclear power but also advances the state of digital twin modeling.

Extensive experiments on datasets from 17 independent nuclear units confirm ETDTN’s superiority over established baselines such as GRU-D, Informer, and Autoformer. The results highlight its particular strength under low-fault-rate scenarios, where subtle anomalies must be detected with high precision to ensure system safety. These findings emphasize the necessity of explicitly modeling temporal variability and latent graph associations in digital twins for nuclear applications.

Although this work focuses on nuclear power, its theoretical framework and practical design can be extended to other critical domains. The proposed ETDTN framework can be extended to other safety-critical domains such as aerospace, smart grids, and industrial manufacturing. Its core modules—continuous-variable temporal representation, latent graph attention, and cloud–edge federated collaboration—are generalizable to any multi-sensor and non-IID monitoring environment. However, practical deployment in different processes would require adaptation of the input feature set, time resolution, and subsystem topology to match the characteristics of each application scenario. Therefore, ETDTN provides a transferable architecture that can be customized for various safety-critical applications rather than a one-size-fits-all solution.

Future research should refine communication-efficient training strategies, optimize edge-level computation, and explore integration with predictive maintenance and real-time decision support systems. Such advances will further enhance the resilience, safety, and sustainability of nuclear power operations and offer a scalable paradigm for digital twin development across diverse industrial ecosystems.

## Figures and Tables

**Table 1:** Fault detection performance test (metric: F1, unit: %).

Baselines	CD			TC			TS			PO		
	f < 0.001	f < 0.01	f < 0.05	f < 0.001	f < 0.01	f < 0.05	f < 0.001	f < 0.01	f < 0.05	f < 0.001	f < 0.01	f < 0.05
PROPHET	59.32	53.28	49.49	72.27	71.81	64.6	71.04	67.39	54.35	47.8	45.24	40.49
GRU-D	64.52	61.3	57.08	70.72	66.18	59.35	74.25	70.19	64.59	60.32	58.13	52.28
VAE-RNN	61.62	58.46	53.29	71.38	67.16	63.47	74.64	73.41	63.28	56.31	53.86	49.70
ODE-RNN-ODE	77.66	81.15	65.77	87.95	83.71	78.64	79.24	84.17	79.82	83.36	80.39	72.12
Informer	80.42	84.9	74.54	83.39	85.35	82.01	91.09	84.49	79.23	87.66	82.94	78.56
Autoformer	85.45	87.18	82.25	80.32	80.59	82.86	83.36	84.20	79.26	81.33	86.10	80.28
ETDTN	92.01	87.99	83.13	96.2	89.32	87.18	92.35	88.24	87.99	89.27	86.27	85.98

**Table 2:** Ablation experiment results (metric: F1, unit: %).

c	CD	TC	TS	PO
TAR-	53.52	44.03	32.12	35.72
TW-	51.15	48.21	37.72	41.85
CVTR-	74.76	71.65	72.49	76.20
ETDTN	87.71	90.90	89.53	87.16

**Table 3:** Parameter analysis.

The Value of *τ*	CD	TC	TS	PO
0	79.8	78.3	78.1	75.2
0.25	82.2	79.4	78.3	76.6
0.5	82.9	80.2	79.2	77.2
0.75	83.2	81.5	80.0	78.8
1	82.0	79.2	78.5	77.4
1.25	81.3	78.7	76.4	76.3

**Table 4:** Training Efficiency and Communication Overhead.

Method	Avg. Time per Round (s)	Uplink + Downlink Payload (MB)
FedAvg	0.42	5.2
FedProx	0.47	5.4
ETDTN	0.63	6.1

## Data Availability

Due to the sensitivity of nuclear power industry data, the datasets used in this study cannot be made publicly available.
